# A hybrid framework combining microwave sensing and deep learning for real-time soil volumetric water content estimation

**DOI:** 10.1038/s41598-026-65948-w

**Published:** 2026-08-14

**Authors:** Adel M. Soliman, Amr Elbrashy, Amr H. Hussien, Ashraf Elmorsy

**Affiliations:** 1 Department of Electronics and Communications, Faculty of Engineering, Horus University-Egypt (HUE), New Damietta, 34517 Egypt; 2 Department of Mechatronics, Faculty of Engineering, Horus University-Egypt (HUE), New Damietta, 34517 Egypt; 3 Department of Electronics and Electrical Communications Engineering, Faculty of Engineering, Tanta University, Tanta, Egypt

**Keywords:** Remote sensing, Volumetric water content (VWC), Convolutional neural network (CNN), Non-destructive testing, Soil moisture., Engineering, Environmental sciences

## Abstract

This study introduces an integrated, non-destructive method for real-time soil moisture measurement using a 2.4 GHz microwave/RF sensing system combined with machine learning. It overcomes the limitations of traditional gravimetric techniques, which are destructive, energy-intensive, and have a 24-hour delay, by providing quick, on-site estimates of volumetric water content (VWC) for urgent geotechnical decisions. The system pairs a 2.4 GHz radar platform with edge computing via a Raspberry Pi 5 to gather and analyze reflected signal power, which correlates strongly with soil dielectric properties. A convolutional neural network (CNN) classifies soil texture (sand, clay, or mixed), allowing for the selection of optimal polynomial regression models for precise prediction. Validation with target water contents of 0.05, 0.15, 0.25, 0.35 and 0.45 L showed high agreement between measured and predicted values, with average accuracies around 99.8%, 99.7%, and 99.4% for sand, clay, and mixed soil, respectively. The system dramatically reduces measurement time to real-time, maintaining reliability compared to the standard gravimetric method, which usually takes 24 h. This approach offers a cost-effective, scalable solution for continuous soil monitoring in civil engineering and agriculture, improving the assessment of foundational stability and enabling proactive risk management.

## Introduction

Soil moisture is a controlling state variable in the mechanical, hydraulic, and environmental behavior of near-surface soils^1^. In geotechnical engineering, variations in volumetric water content influence matric suction, shear strength, compressibility, shrink–swell response, bearing capacity, and slope stability, thereby affecting the performance of foundations, pavements, embankments, retaining structures, and compacted barriers^2^. In agricultural and environmental systems, soil moisture governs infiltration, evapotranspiration, irrigation scheduling, root-zone water availability, runoff generation, and land–atmosphere exchange processes. Consequently, accurate and timely determination of volumetric water content (VWC) is essential for risk-informed decision-making in both infrastructure and water-resource management applications^3^. Recent advances in decentralized solar–water and solar–food systems further show that water-resource management is increasingly linked with clean-energy conversion, resource recovery, and autonomous field operation, reinforcing the need for rapid and deployable soil-moisture monitoring tools in agricultural and environmental applications^4–6^. Over the past two decades, the evolution of remote sensing technologies has transitioned from labor-intensive, point-based in-situ measurements to highly scalable, non-destructive electromagnetic (EM) and radar sensing techniques^7,8^.

Recent studies have increasingly shifted soil moisture assessment from conventional destructive procedures toward rapid, non-destructive electromagnetic and radar-based sensing techniques. Huang, et al.^9^ demonstrated that compact continuous-wave radar systems can estimate soil moisture by analyzing radar echo characteristics, confirming the potential of radar signals as moisture-sensitive indicators. Similarly, Pathirana, et al.^10^ showed that electromagnetic and ground-penetrating radar measurements can provide reliable information about shallow soil water content, supporting the use of geophysical sensing as an alternative to labor-intensive point measurements. These studies confirm the suitability of radar and electromagnetic methods for non-destructive soil moisture monitoring. However, most existing radar-based approaches remain limited by site-specific calibration requirements, sensitivity to soil properties, and insufficient integration with real-time embedded processing.

Machine learning has also become a major tool for improving the interpretation of electromagnetic and radar signals in soil moisture estimation^11^. Vahidi, et al.^12^ showed that convolutional neural networks can extract moisture-sensitive patterns from ground-penetrating radar data and improve prediction accuracy at different soil depths. Progga, et al.^13^ further demonstrated that machine learning and deep learning models can improve the conversion of radio-frequency sensor responses into volumetric moisture content estimates. Wan, et al.^14^ also confirmed that particle-size distribution and soil physical properties are important predictors in machine-learning-based estimation of soil water content. These findings indicate that data-driven models can manage nonlinear relationships between sensor signals and soil moisture more effectively than simple empirical equations. Nevertheless, many existing machine-learning approaches depend on complex sensing platforms, offline processing, or generalized calibration models, which may reduce their practical suitability for continuous real-time field monitoring. This limitation is also consistent with the broader transition toward mechatronic systems in which sensing, control, AI, and IoT are integrated to improve real-time monitoring, operational resilience, and autonomous decision-making in sustainable engineering applications^15^.

A major limitation identified in the literature is the lack of transferability of soil moisture prediction models across different soil textures^16^. Awais, et al.^17^ emphasized that dielectric-based moisture estimation is strongly affected by soil texture, mineral composition, salinity, electrical conductivity, and other physical characteristics. Similarly, Szypłowska, et al.^18^ showed that the accuracy of volumetric water content prediction can be improved when soil-specific correction factors are incorporated into dielectric calibration models. These studies confirm that a universal calibration model may not provide reliable results for soils with contrasting textures such as sand, loam, and clay. However, most previous studies focused either on developing calibration equations or improving sensor response interpretation, without integrating automatic soil texture classification with texture-specific moisture prediction. This creates a clear need for adaptive frameworks that first identify soil type and then apply the most suitable prediction model.

From an application perspective, real-time soil moisture estimation is particularly important in geotechnical engineering, agriculture, and environmental monitoring. Piciullo, et al.^19^ showed that volumetric water content, pore-water pressure, rainfall, and vegetation-related variables can support short-term slope stability forecasting. Tan and Vanapalli^20^ demonstrated that rainfall infiltration, matric suction variation, groundwater depth, soil type, slope angle, and foundation location significantly influence the bearing capacity of shallow foundations on unsaturated slopes. These findings confirm that accurate soil moisture monitoring is essential for assessing foundation stability, slope safety, and moisture-induced ground deterioration. However, existing geotechnical monitoring approaches often rely on embedded sensors, numerical models, or complex monitoring networks, while conventional gravimetric testing remains destructive, time-consuming, and unsuitable for real-time decision-making. Alternative high-resolution monitoring frameworks, such as Actively Heated Fiber Optic (AHFO) sensor arrays^21,22^, offer continuous thermal-conductivity based profiling along critical geotechnical infrastructures like slopes or earth dams. While AHFO systems provide superior continuous spatial profiling, they entail highly invasive subsurface trenching, complex distributed interrogation hardware, and significant energy footprints for thermal injection. In contrast, the microwave/RF edge-processing approach presented herein operates on an entirely distinct dielectric measurement principle. It offers a non-destructive, ultra-low-cost, and highly portable alternative optimized for single-point spot-checking applications where invasive infrastructure deployment or permanent fiber embedding is unfeasible.

### Research gap

In the lifecycle of large-scale civil engineering projects, geotechnical stability serves as the primary determinant of structural longevity. Soil moisture monitoring is not merely a secondary environmental metric but a critical variable in foundational design and the mitigation of long-term structural risks. However, industry-standard methodologies for assessing soil water content have remained largely stagnant, failing to keep pace with the compressed timelines of modern construction. As projects shift toward smarter, IoT-integrated infrastructure, the latency between field sampling and laboratory results creates a significant strategic bottleneck, leading to potential errors in foundational loading or overlooked risks during soil compaction.

### Paper contributions

Leveraging electromagnetic sensing and image processing, the presented paper proposes a framework where the type of soil categorized broadly into clay, sand, and mixed soil is first classified in real time using a lightweight machine learning classifier trained on a large number of different soil images, employing the convolutional neural network (CNN). Once the soil type of the sample under testing is accurately identified, the system dynamically selects the optimal, pre-calibrated 4th-degree polynomial regression coefficients tailored specifically to that soil category. The system is decomposed into two parallel sensing pipelines. First, a radar-based moisture estimation pipeline captures subsurface-sensitive electromagnetic reflections, and additionally extracts signal features correlated with dielectric properties and maps reflected power to $\:{\theta\:}_{v}$. Secondly, the vision-based soil classification pipeline captures surface texture and color features.

This paper adopts a hybrid experimental computational research design to develop and validate a non-invasive soil characterization system that integrates electromagnetic sensing with artificial intelligence. The primary objectives of the proposed framework are:

1. To quantitatively estimate soil moisture content using radar backscatter analysis.
2. To qualitatively classify soil type using deep learning-based image analysis.
3. To develop a data fusion strategy that improves reliability across heterogeneous soil conditions.

## Materials and methods

This section describes the experimental setup, detailing the soil sample preparations (Sand, Clay, and Mixed), the hardware integration of the 2.4 GHz radar with the Raspberry Pi 5 platform, and the mathematical framework for calculating Volumetric Water Content (VWC). Furthermore, it outlines the dual-stage processing pipeline and explains the implementation of the CNN for soil classification and the subsequent application of polynomial regression models for moisture inversion.

### Theoretical framework

**Fig. 1:**
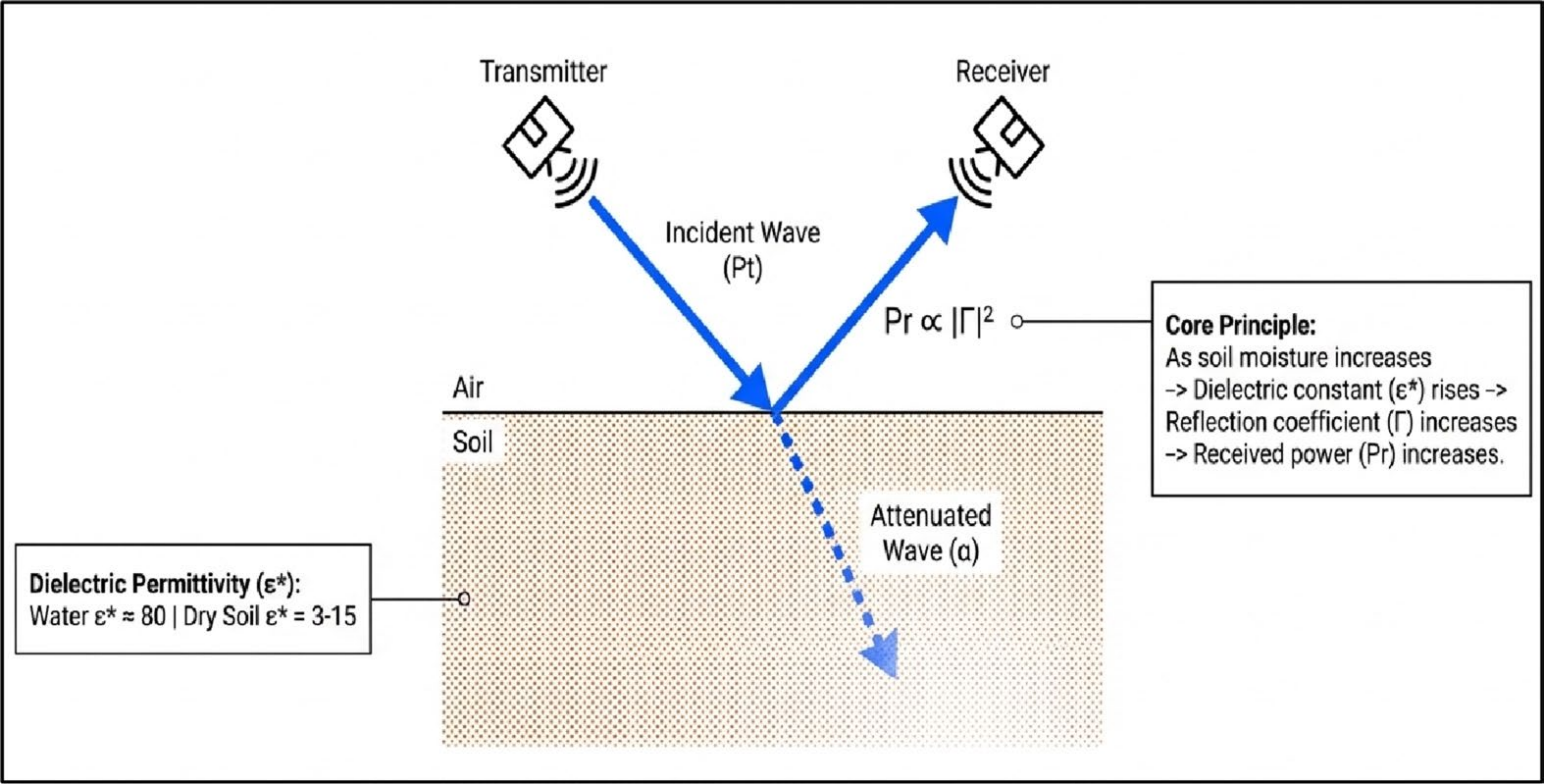
Principle of operation of radar-based VWC measurement system.

The principle of operation of the proposed radar-based VWC measurement system is based on the interaction between incident electromagnetic (EM) waves and the soil medium. When a wave propagating in air encounters the soil surface, part of the energy is reflected while the remainder is transmitted and attenuated within the soil as shown in Fig. 1.

The behavior of the wave is governed by the **complex dielectric permittivity** $\:{\epsilon\:}^{*}$of soil that is defined as^11,23^:

1 $$\:{\epsilon\:}^{*}={\epsilon\:}^{{\prime\:}}-j{\epsilon\:}^{{\prime\:}{\prime\:}}$$

Where $\:{\epsilon\:}^{{\prime\:}}$ is the real part of the dielectric constant, that represents the energy storage. While $\:{\epsilon\:}^{{\prime\:}{\prime\:}}$is the imaginary part of the dielectric constant that represents the energy dissipation. Soil is a heterogeneous medium composed of solid particles, air voids, and water, since water has a significantly higher dielectric constant (~ 80) compared to dry soil (~ 3–5), even small variations in moisture content lead to measurable changes in $\:{\epsilon\:}^{*}$. The volumetric water content $\:{\theta\:}_{v}$ is strongly correlated with the dielectric constant via the empirical model (like the Topp model) defined below^18,23^.

2 $$\:{\epsilon\:}^{{\prime\:}}={a}_{0}+{a}_{1}{\theta\:}_{v}+{a}_{2}{\theta\:}_{v}^{2}+{a}_{3}{\theta\:}_{v}^{3}$$

where $\:{a}_{i},\:i=0,\:1,\:2,\:3$ are soil-dependent coefficients. This nonlinear relationship forms the theoretical basis for radar-based moisture estimation.

At the air–soil interface, the reflection of the incident wave is described by the Fresnel equations. For perpendicular polarization, the reflection coefficient $\:{\Gamma\:}$ is given by^23,24^.

3 $$\:{\Gamma\:}=\frac{\sqrt{{\epsilon\:}^{*}}\mathrm{c}\mathrm{o}\mathrm{s}{\theta\:}_{i}-\sqrt{1-{\mathrm{s}\mathrm{i}\mathrm{n}}^{2}{\theta\:}_{i}}}{\sqrt{{\epsilon\:}^{*}}\mathrm{c}\mathrm{o}\mathrm{s}{\theta\:}_{i}+\sqrt{1-{\mathrm{s}\mathrm{i}\mathrm{n}}^{2}{\theta\:}_{i}}}$$

where $\:{\theta\:}_{i}$ is the incidence angle. The reflected power ($\:{P}_{r}$) is proportional to the square of the magnitude of the reflection coefficient^23,24^.

4 $$\:{P}_{r}\propto\:\mid\:{\Gamma\:}{\mid\:}^{2}$$

Therefore, as soil moisture increases, $\:{\epsilon\:}^{*}$ increases, leading to a stronger reflected signal detected by the radar. So, for practical implementation, the physical model is reduced to an empirical relationship^13,25^

5 $$\:{P}_{r}=f\left({\theta\:}_{v}\right)$$

Guided by the established dependence of soil dielectric behavior on water content and texture, an empirical soil-specific polynomial regression model was fitted to the measured RSSI–moisture calibration data^11,25^:

6 $$\:{\theta\:}_{v}={\beta\:}_{4}{RSSI}^{4}+{\beta\:}_{3}{RSSI}^{3}+{\beta\:}_{2}{RSSI}^{2}+{\beta\:}_{1}\mathrm{R}\mathrm{S}\mathrm{S}\mathrm{I}+{\beta\:}_{0}$$

where $\:{\theta\:}_{v}$ is the dependent variable representing the estimated soil moisture, RSSI is the independent variable (raw sensor input), and $\:{\beta\:}_{0},\:{\beta\:}_{1}.\:{\beta\:}_{2}.\:{\beta\:}_{3},$ and $\:{\beta\:}_{4}$ are the regression coefficients unique to the specific soil dielectric properties. The system evaluates the goodness-of-fit using the coefficient of determination ($\:{R}^{2}$).

The selection of a 4th-degree polynomial regression model for mapping raw RSSI measurements to VWC is predicated on the need to characterize the complex, non-linear dielectric c response of soil while maintaining computational efficiency for edge deployment. The physical relationship between soil dielectric permittivity and moisture content is inherently non-linear, often exhibiting distinct inflection points influenced by soil texture, porosity, and bulk density. While lower-order models often fail to capture these higher-order non-linearities, resulting in systematic underfitting, excessively high-order polynomials increase the risk of overfitting, particularly in the presence of sensor noise. A 4th-degree polynomial provides the optimal balance: it offers sufficient degrees of freedom to capture the nuanced, site-specific dielectric characteristics of heterogeneous soil samples (clay, sand, and mixed) while maintaining a monotonic behavior within the practical range of VWC measurements.

The dataset was split into 70% for training and 30% for validation. This allowed for continuous assessment of the model’s generalization capacity during training and provided insight into the effectiveness of the chosen architecture and optimization strategy. The training setup was implemented using TensorFlow and Keras frameworks in a Python environment, ensuring scalability and compatibility with deployment on the Raspberry Pi system. To assess the effectiveness of the proposed CNN-based soil classification model, a comprehensive evaluation was conducted using a range of performance metrics derived from both the training and validation phases. The model was trained for 30 epochs (An epoch refers to one complete pass through the entire training dataset. In this case, the model underwent 30 such passes to iteratively learn patterns from the data) using the Adam optimizer, a learning rate of 0.001, and a batch size of 16. These parameters were selected to achieve a balance between training speed and convergence stability, particularly considering the limited computational resources of edge devices such as the Raspberry Pi. There are four potential results for the matrix: Truly Positive (TP), False Positive (FP), Truly Negative (TN), and False Negative (FN). The following equations represent evaluation matrices applied in the proposed system.

Accuracy: the proportion of all classifications that were correct, whether positive or negative, the model accuracy can be determined using (Eq. 7)^26^.

7 $$\:Accuracy=\frac{TP+\:TN}{TP+\:FP+\:TN+\:FN}$$

Precision: It provides an indication of the cases that have proved to be positively correct. The precision can be determined using (Eq. 8).

8 $$\:Precision=\:\frac{TP}{TP+\:FP}\:$$

Recalling or True Positive Rate (TPR): This will give an idea of whether the applied model is accurate or not. The recall can be determined by using (Eq. 9).

9 $$\:Recall=\:\frac{TP}{TP+\:FN}$$

F1-score: F1-score offers a summary of precision and recall. It can be determined by using (Eq. 10).

10 $$\:F1\:Score=\:\frac{2}{\left(\:\raisebox{1ex}{$1$}\!\left/\:\!\raisebox{-1ex}{$Recall$}\right.\right)\:+\:\:\left(\raisebox{1ex}{$1$}\!\left/\:\!\raisebox{-1ex}{$Precision$}\right.\:\right)\:}$$

Specificity or True Negative Rate (TNR): It can be calculated by using (Eq. 11).

11 $$\:TNR=\:\frac{TN}{TN+FP}$$

### System architecture

**Fig. 2:**
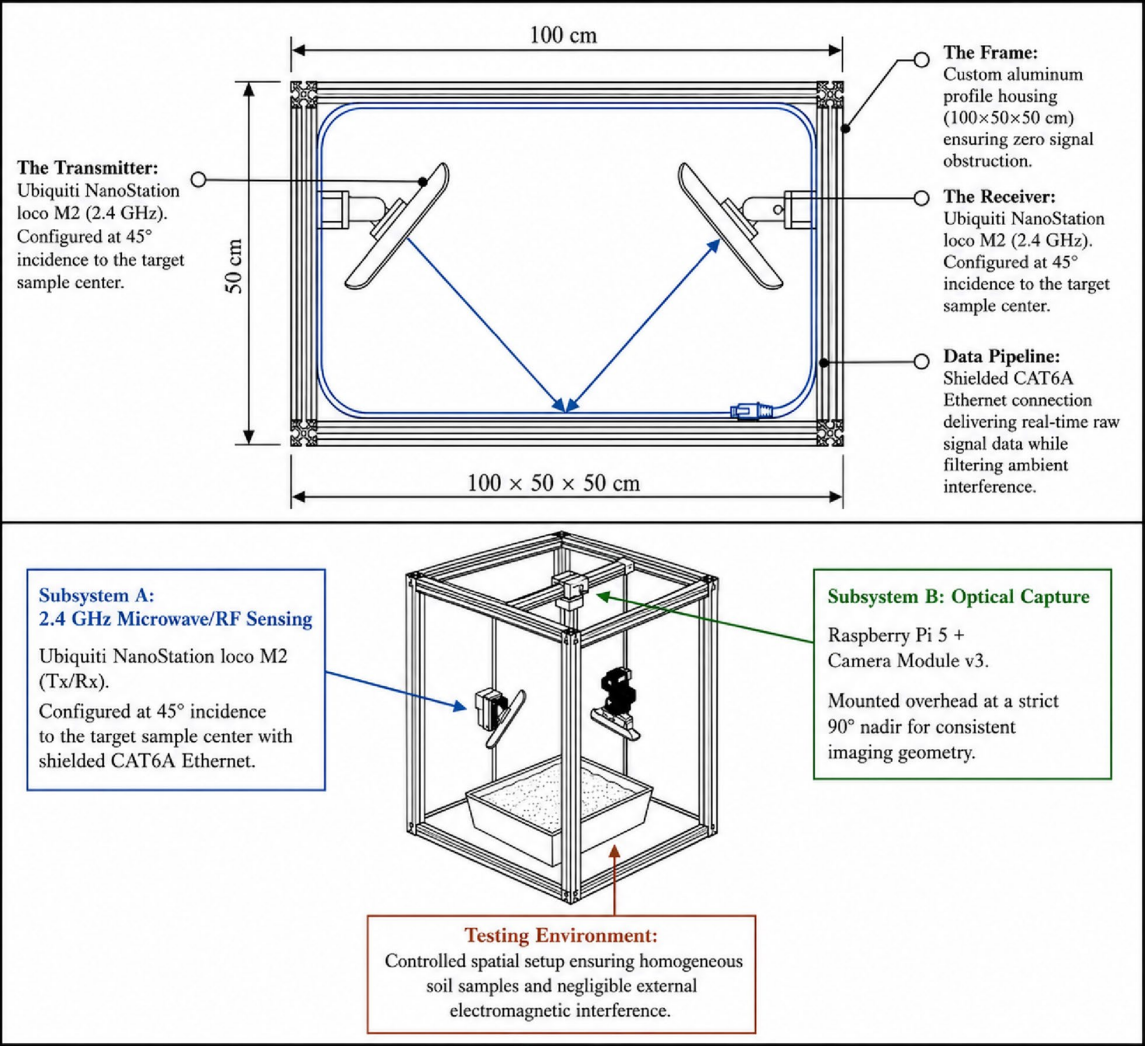
Proposed System Architecture.

The proposed system architecture comprises two integrated subsystems designed to enable real-time soil analysis through automated soil classification and non-destructive moisture estimation as shown in Fig. 2. These subsystems operate sequentially to ensure that soil type identification informs the selection of the appropriate moisture prediction model. The radar subsystem is responsible for estimating soil volumetric water content (VWC) using a non-contact sensing approach. It follows a linear signal-processing workflow consisting of transmission, reflection, reception, and data processing stages. Initially, a transmitter emits a 2.4 GHz electromagnetic signal toward the soil surface. The signal interacts with the soil medium, where its reflection characteristics vary according to the soil’s dielectric properties. The receiver captures the reflected signal, which is then processed to extract reflection power and estimate moisture content. The components are mounted on a lightweight aluminum frame (100 cm × 50 cm × 50 cm) that ensures structural stability while allowing flexible access for calibration and testing. The hardware configuration includes two Ubiquiti NanoStation M2 LOCO devices functioning as transmitter and receiver, both equipped with directional antennas to enhance signal focus and measurement accuracy. Data transmission and power supply are achieved using shielded CAT6A Ethernet cables, minimizing electromagnetic interference. A custom graphical user interface (GUI) running on a connected computer provides real-time visualization of signal metrics, including reflection power, signal strength, and noise levels, and computes the final moisture output.

### System initialization and laboratory calibration

**Fig. 3:**
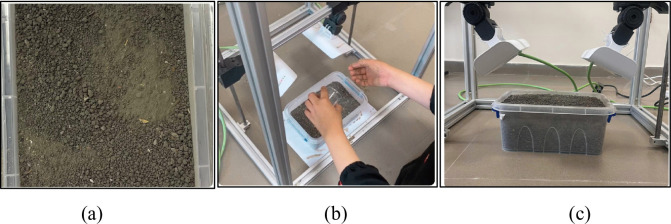
(a) prepared dried sample, (b) Incremental hydration, and (c) Electromagnetic measurement.

The framework begins with the definition of the physical and operational parameters of the radar/RF system. Key parameters include the operating frequency (set at 2.4 GHz), the link distance between the transmitter and receiver, and the antenna gain, all of which dictate the baseline free-space path loss and signal penetration capabilities. To establish an empirical baseline, a controlled laboratory analysis is conducted for each target soil type using the following three steps, shown in Fig. 3.

i. *Controlled Desiccation*: Soil samples are first completely dried to represent a 0% VWC baseline.
ii. *Incremental Hydration*: Water content is increased in controlled, uniform steps (+ 5%).
iii. *Electromagnetic Measurement*: At each step, the RSSI is measured in Watt. Because soil moisture drastically alters the dielectric constant of the medium, the RSSI serves as a direct, observable proxy for signal attenuation caused by the water molecules.

Soil texture profoundly affects its water-holding capacity and dielectric boundary behaviors. Therefore, a static equation yields massive errors. The right side of the flowchart illustrates a dynamic, adaptive execution branch:

i. *Subsystem Decision*: The system checks if a localized soil classification is available (fed by an auxiliary Convolutional Neural Network (CNN) subsystem that analyzes ground imagery or radar return profiles).
ii. *Default “Universal” Mode*: If no classification is active, the algorithm defaults to a “Universal” polynomial (optimized for generic loam) using pre-stored universal coefficients.
iii. *Class-Specific Branching*: If the CNN successfully classifies the soil, the algorithm branches based on the detected physical medium:

- Clay: High dielectric sensitivity; specific stored coefficients are selected.
- Sand: Low dielectric sensitivity/high drainage; specific stored coefficients are selected.
- Mixed Soil: If the medium is a mixture, the system checks for a known mix ratio. If known, it computes a Weighted Average of the polynomial coefficients to generate a custom curve. If unknown, it falls back to the universal model.

The second subsystem performs soil type classification using a computer vision-based approach to ensure accurate selection of the moisture estimation model. The processing pipeline follows a structured flow: image acquisition, preprocessing, feature extraction, classification, and output display. A Raspberry Pi Camera Module v3 with a 12 MP sensor captures high-resolution images of the soil surface. These images are transmitted via a 22-pin CSI interface to a Raspberry Pi 5, which serves as the edge processing unit. The system runs a pre-trained convolutional neural network (CNN) model that performs feature extraction through convolutional and pooling layers, followed by classification using fully connected layers. The model categorizes soil into predefined classes such as clay, sand, and mixed soil types. The predicted class is displayed in real time, with a confidence threshold applied to ensure reliability; predictions below 70% confidence are labeled as “unknown.” This classification output is subsequently used to select the appropriate regression model for accurate soil moisture estimation. To ensure real-time execution on resource-constrained edge hardware without sacrificing classification accuracy, the vision subsystem employs a customized MobileNetV3-Small backbone. This architecture was selected specifically for its optimal balance between computational efficiency and feature-extraction capability, utilizing depthwise separable convolutions and squeeze-and-excitation modules to minimize the mathematical operations required per inference cycle. For deployment on the edge controller (Raspberry Pi 5), the trained PyTorch/TensorFlow model was frozen and compiled into a highly optimized TensorFlow Lite format. This compilation process leverages post-training quantization, mapping 32-bit floating-point weights to 8-bit integers, which drastically reduces memory consumption and accelerates matrix multiplication operations on ARM-based CPU architectures. Extensive profiling of the deployed model demonstrates exceptional suitability for edge computing. The finalized architecture contains approximately 1.5 million trainable parameters, necessitating a highly compressed on-device memory footprint of just 4.2 MB. In terms of computational complexity, the model requires only ~ 0.05 Giga Floating-Point Operations (GFLOPs) per forward pass. During rigorous on-device benchmarking under standard operating temperatures, the vision pipeline achieved an average inference latency of 12.4 ± 1.2 ms. This millisecond-level latency guarantees that the soil classification acts as a true real-time feed-forward layer, introducing negligible delay before dynamically selecting the appropriate polynomial regression model for the radar-based moisture estimation.

To ensure the optical classification pipeline remains robust against ambient environmental disturbances during field deployment, the vision hardware is integrated into a rigid, light-shielded structural enclosure. This shrouded design physically locks the Raspberry Pi Camera at a strict 90-degree nadir viewing geometry and actively mitigates severe variations in external solar illumination or shadowing. By standardizing the physical optical environment, the enclosure ensures that field-acquired imagery closely mirrors the geometric and lighting consistency of the controlled laboratory setup. Furthermore, to bridge the gap between ideal laboratory conditions and potential field-site anomalies, the CNN training framework incorporated a comprehensive data augmentation pipeline. Prior to feature extraction, the training dataset was artificially expanded using random brightness scaling (**± 20%**), contrast adjustments, Gaussian noise injection, and synthetic shadow overlays. This proactive robustification exposes the neural network to a wide spectrum of visual disturbances, ensuring the classifier remains invariant to residual lighting dynamics, minor surface roughness, or stray scattering effects that might bypass the physical enclosure in real-world scenarios.

The finalized database contains 1,200 unique macro-photographs equally distributed across the three target classes (400 images per class) captured at a resolution of $\:1920\times\:1080$ pixels using a fixed 15 cm focal distance and 90-degree nadir angle under stabilized ambient conditions. To enhance model generalizability and prevent overfitting to lab settings, this base dataset was expanded to 4,800 matrices using the aforementioned data augmentation pipeline consisting of random geometric transformations, contrast stretching, and synthetic shadow overlays.

To prevent the convolutional neural network from overfitting to superficial chromatic profiles (soil color) rather than authentic morphological properties, a rigorous image preprocessing pipeline was enforced. Prior to training, all input images are converted to single-channel grayscale and subjected to adaptive histogram equalization. This technical step suppresses raw color signatures and forces the network to extract invariant geometric features, such as structural edges, granular distributions, and textural shadows. Consequently, the vision subsystem achieves robust classification invariance against shifting mineralogies or moisture-induced soil darkening.

**Fig. 4:**
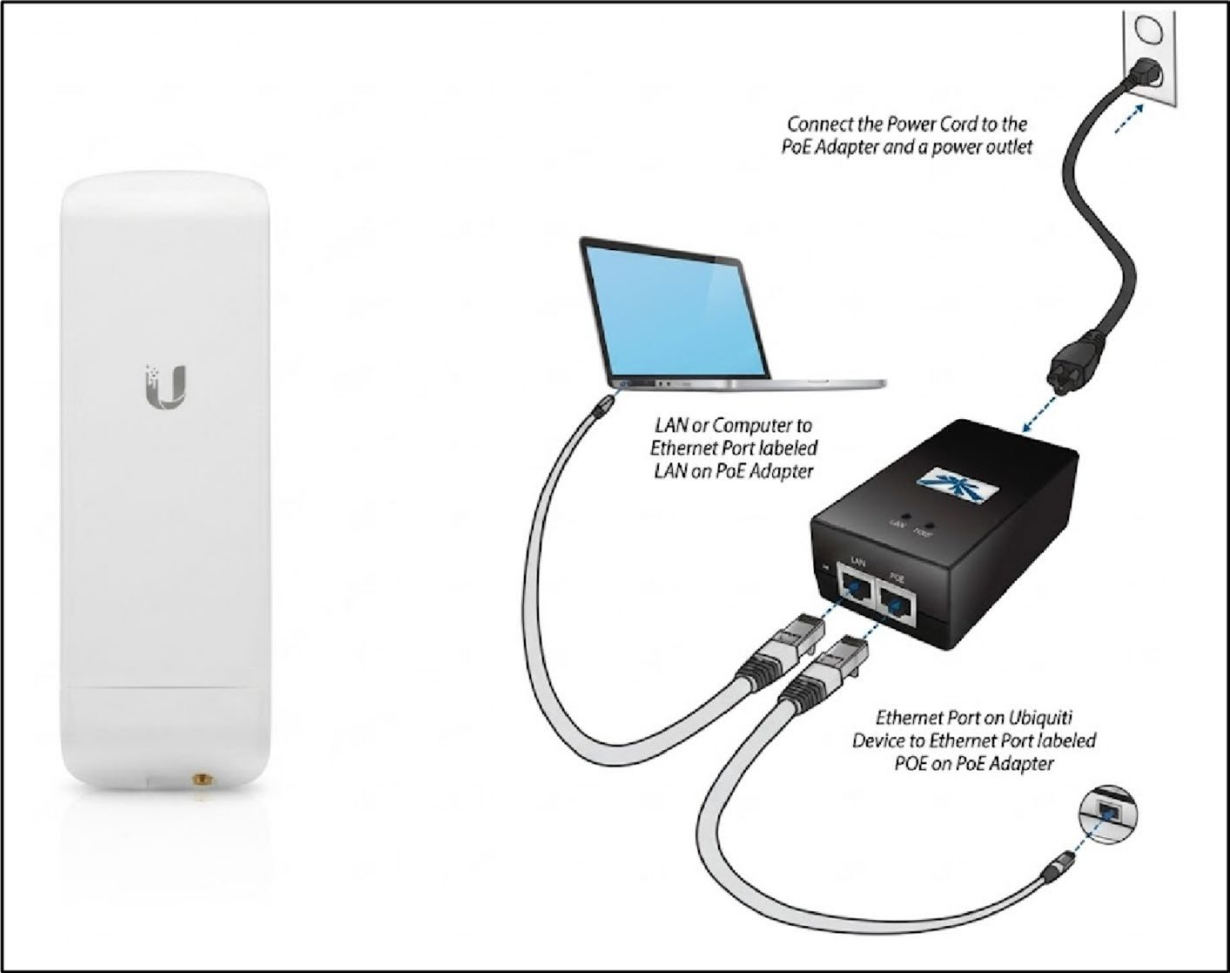
NanoStation M2 LOCO connection setup.

The communication architecture of the proposed radar-based soil moisture measurement system utilizes the Ubiquiti NanoStation M2 LOCO shown in Fig. 4. This compact wireless transponder is engineered for long-range outdoor signal transmission and serves as a primary link for high-reliability data backhaul over extended distances.

The device operates within the 2.4 GHz Industrial, Scientific, and Medical (ISM) band. While this frequency offers superior propagation characteristics over long distances, the system design accounts for potential electromagnetic interference from co-located consumer electronics, such as mobile devices and microwave appliances. To mitigate these effects and optimize throughput in high-density environments, the system leverages Ubiquiti’s proprietary airMAX technology. This Time Division Multiple Access (TDMA) protocol minimizes collision-induced latency and enhances overall network efficiency.

Signal directionality is maintained via an integrated 60-degree directional antenna with a gain of 10 dBi. This focused beamwidth reduces the impact of neighboring network interference and facilitates stable communication links at ranges exceeding 5 km, contingent upon environmental variables and Fresnel zone clearance.

The internal processing unit is based on a MIPS 32-bit architecture, supported by 64 MB of RAM and 8 MB of non-volatile flash memory for firmware and configuration storage. This hardware profile ensures consistent data traffic handling without performance degradation during intensive soil moisture data acquisition. Physical deployment is streamlined through an Ethernet-based Power over Ethernet (PoE) configuration, which consolidates data transmission and DC power delivery into a single cable interface.

**Fig. 5:**
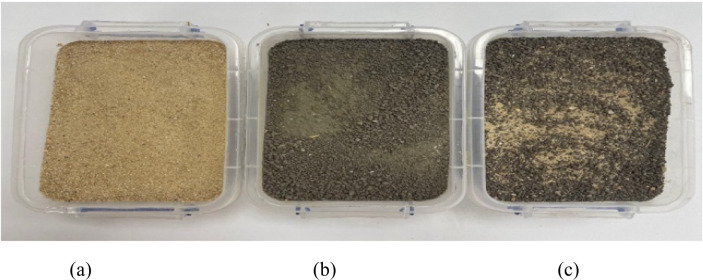
Soil samples (a) Sand, (b) Clay, (c) mixed.

The experimental validation of radar-based VWC estimation requires a rigorous protocol for soil sample preparation to ensure that dielectric responses are attributable solely to moisture variations. In this paper, three distinct soil textures were selected based on their contrasting electromagnetic and hydraulic properties: sandy soil, clay soil, and a heterogeneous mixture of both, classified as sandy clay as shown in Fig. 5. These samples represent a broad spectrum of density, porosity, and moisture retention behaviors, which are critical factors in calibrating the 4th-degree polynomial regression coefficients used for moisture inversion. The dielectric constant of soil is a function of its physical composition and chemical environment. Sandy soil, characterized by a coarse texture and a high sand content of 85%, exhibits a high bulk density $\:\left(\approx\:1.6\:\mathrm{g}/{\mathrm{c}\mathrm{m}}^{3}\right)$ and low organic matter (0.5–1.0%), leading to rapid drainage and a low wilting point of 3.3%. Conversely, clay soil (60% clay content) features a fine texture with a lower density range $\:\left(1.1-1.3\:\mathrm{g}/{\mathrm{c}\mathrm{m}}^{3}\right)$ but significantly higher water retention capacity, evidenced by a wilting point of 27.2% and maximum moisture levels reaching 40%. The composite ‘mixed soil’ class was formulated as a heterogeneous matrix texturally classified as sandy clay, establishing an intermediate dielectric environment via a balanced, uniform composition of sand, silt, and clay. To guarantee matrix homogeneity and strictly avoid granular segregation across sequential experimental runs, the dry mixture was processed using a mechanical laboratory rotary blender for 30 min. Following blending, the composite was normalized by passing it through a standard No. 10 sieve mesh (2.0 mm opening) to achieve uniform aggregate distribution prior to moisture calibration. The detailed physical and chemical properties of these substrates are summarized in Table 1.

**Table 1:** Physical and chemical properties of soil samples.

Soil Type	Sand/Silt/Clay (%)	Density (g/cm3)	Wilting Point (%)	Moisture Content (%)	Texture Class	pH Range	Organic Matter (%)
Sandy	85/10/5	1.6	3.3	10–12	Coarse	5.5–6.5	0.5–1.0
Clay	20/20/60	1.1–1.3	27.2	30–40	Fine	6.5–7.5	2.0–4.0

### System workflow

**Fig. 6:**
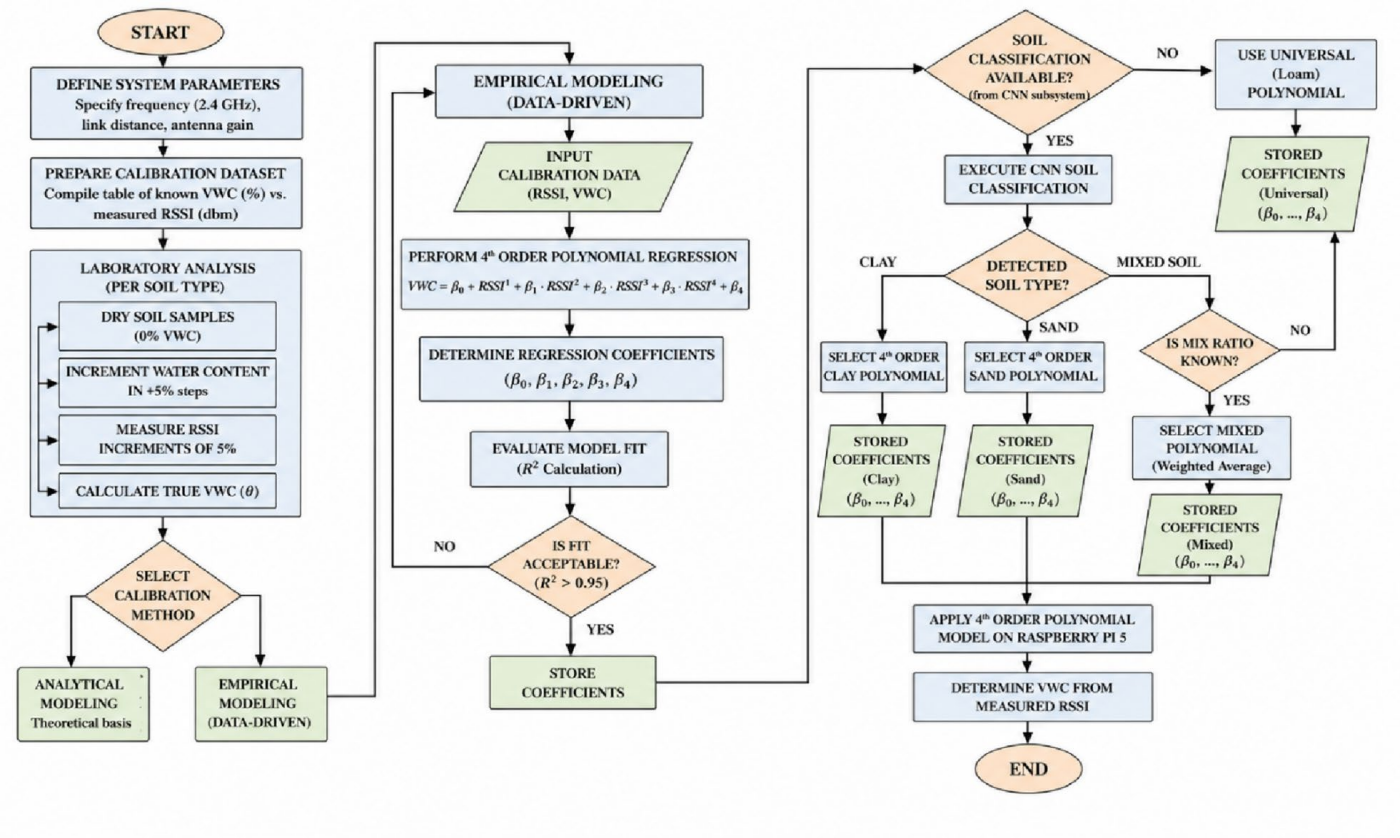
The flowchart of the proposed radar-based RSSI system for VWC measurement.

The proposed system workflow shown in Fig. 6 outlines a data-driven framework for estimating VWC using radar-based Received Signal Strength Indicator (RSSI) measurements across various soil textures. It employs a standardized preparation protocol and 4th-degree polynomial regression to establish soil-specific calibration coefficients. An integrated CNN subsystem enables real-time soil classification, allowing the system to adaptively select or weigh these coefficients for precise moisture determination on an edge computing device, as described below.

## Results and Discussion

This section outlines the performance of the proposed system, organized into two primary analytical phases. The first phase establishes a calibration framework to define the mathematical relationship between RSSI and VWC across various soil types. The second phase assesses the efficacy of a CNN model in classifying these soil samples. Together, these analyses demonstrate the system’s ability to accurately categorize soil conditions, which serves as a foundational step for precise moisture content estimation.

### Calibration phase

**Fig. 7:**
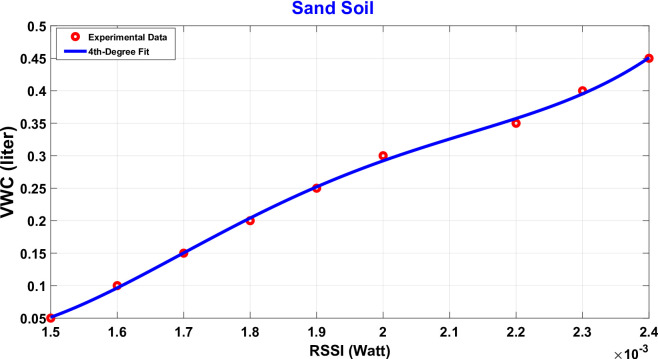
Calibration curve for sand soil.

**Fig. 8:**
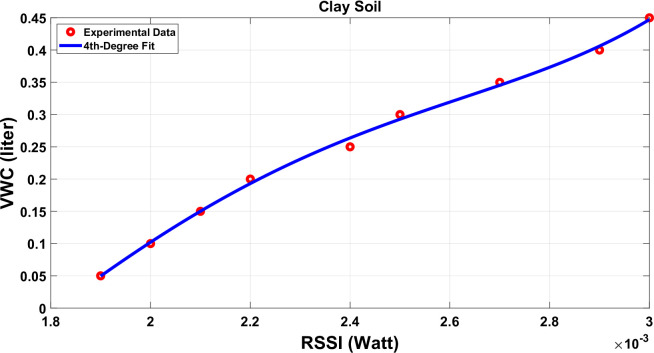
Calibration curve for clay soil.

**Fig. 9:**
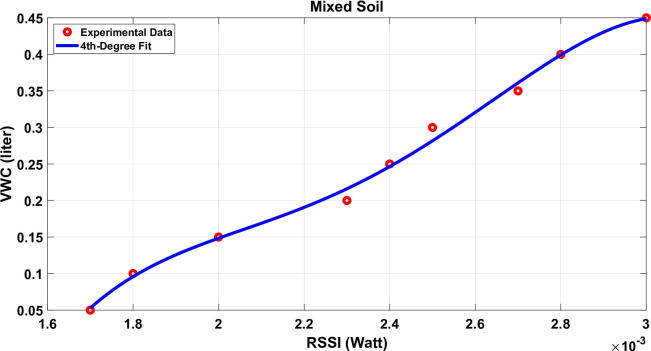
Calibration curve for mixed soil.

The objective of the calibration phase is to establish a precise mathematical correlation between the measured RSSI (mW) and the VWC for three soil types: sand, clay, and mixed soil. A 4th-degree regression model is employed to characterize this relationship. The performance of this model is evaluated against a predetermined threshold for the coefficient of determination $\:\left({R}^{2}>94\%\right)$, which serves as the primary statistical metric for quantifying the goodness of fit between the regression model and the experimental datasets. The experimental datasets, encompassing controlled VWC increases and corresponding RSSI measurements for sand, clay, and mixed soil (respectively shown in Tables 2 and 3, and 4), provided the empirical foundation for this analysis. Based on these inputs, the calibration curves are generated (Figs. 7 and 8, and 9), respectively, visually confirming the accuracy of the 4th-degree polynomial fit.

**Table 2:** Experimental dataset for sand soil.

* VWC*	0.05	0.1	0.15	0.2	0.25	0.3	0.35	0.4	0.45
$\:RSSI\:\left(mW\right)$	1.5	1.6	1.7	1.8	1.9	2.0	2.2	2.3	2.4

**Table 3:** Experimental dataset for clay soil.

* VWC*	0.05	0.1	0.15	0.2	0.25	0.3	0.35	0.4	0.45
$\:RSSI\:\left(mW\right)$	1.9	2.0	2.1	2.2	2.4	2.5	2.7	2.9	3

**Table 4:** Experimental dataset for mixed soil.

* VWC*	0.05	0.1	0.15	0.2	0.25	0.3	0.35	0.4	0.45
$\:RSSI\:\left(mW\right)$	1.7	1.8	2.0	2.3	2.4	2.5	2.7	2.8	3

The derived regression coefficients $\:{\beta\:}_{0}$ through $\:{\beta\:}_{4}$ and the resulting goodness-of-fit metrics $\:\left({R}^{2}\right)$ for each soil type are presented in Table 5.

**Table 5:** Calculated regression coefficients and goodness of fit for tested soil types.

Soil type Coefficients		Sand	Clay	Mixed
4th degree regression coefficients	$\:{\beta\:}_{0}$	* 16.9765*	* 2.734*	* -14.2301*
	$\:{\beta\:}_{1}$	$\:-3.7827\times\:{10}^{4}$	$\:-7.1026\times\:{10}^{3}$	$\:2.4897\times\:{10}^{4}$
	$\:{\beta\:}_{2}$	$\:3.0827\times\:{10}^{7}$	$\:5.7238\times\:{10}^{6}$	$\:-1.6202\times\:{10}^{7}$
	$\:{\beta\:}_{3}$	$\:-1.0889\times\:{10}^{10}$	$\:-1.8388\times\:{10}^{9}$	$\:4.6662\times\:{10}^{9}$
	$\:{\beta\:}_{4}$	$\:1.4235\times\:{10}^{12}$	$\:2.1178\times\:{10}^{11}$	$\:-4.9607\times\:{10}^{11}$
coefficient of determination	$\:{R}^{2}$	$\:99.76\%$	$\:99.7601\mathrm{\%}$	$\:99.4937\mathrm{\%}$

As demonstrated by the analytical results, the 4th-degree polynomial model provides a robust fit for the experimental data across all tested soil compositions. The calculated $\:{R}^{2}$ values ($\:99.76\%$ for sand, $\:99.7601\mathrm{\%}$ for clay, and $\:99.4937\mathrm{\%}$ for mixed soil) consistently surpass the 95% threshold requirement. These high correlation values validate the efficacy of the proposed model, confirming its suitability for accurately estimating $\:VWC={\theta\:}_{v}$ based on observed RSSI measurements using the 4th degree polynomial regression model (Eq. 12). Since each soil type has distinct regression coefficients as listed in Table 5, the VWC for sand, clay, and mixed soil is estimated using Eqs. (13), (14), and (15), respectively^9,11,26^.

12 $$\:{\theta\:}_{v}=\:{\beta\:}_{4}\:{RSSI}^{4\:}+{\beta\:}_{3}\:{RSSI}^{3\:}+{\beta\:}_{2}{\:RSSI}^{2\:}+{\beta\:}_{1}\:RSSI+{\beta\:}_{0}$$

13 $$\:{\left.{\theta\:}_{v}\right|}_{Sand}=\:1.4235\times\:{\:10}^{12}\times\:{RSSI}^{4\:}-1.0889\times\:{10}^{10}\times\:{RSSI}^{3\:}+3.0827\times\:\:{10}^{7}\:\times\:{RSSI}^{2\:}-3.7827\times\:{10}^{4}\times\:\:RSSI+16.9765$$

14 $$\:{\left.{\theta\:}_{v}\right|}_{Clay}=2.1178\times\:{10}^{11}\times\:{RSSI}^{4\:}-1.8388\times\:{10}^{9}\times\:{RSSI}^{3\:}+5.7238\times\:{10}^{6}\times\:{RSSI}^{2\:}-7.1026\times\:{10}^{3}\times\:RSSI+2.734$$

15 $$\:{\left.{\theta\:}_{v}\right|}_{Mixed}=-4.9607\times\:{10}^{11}\times\:{RSSI}^{4\:}+4.6662\times\:{10}^{9}\times\:{RSSI}^{3\:}-1.6202\times\:{10}^{7}\times\:{RSSI}^{2\:}+2.4897\times\:{10}^{4}\times\:{RSSI}^{\:}-14.2301$$

**Fig. 10:**
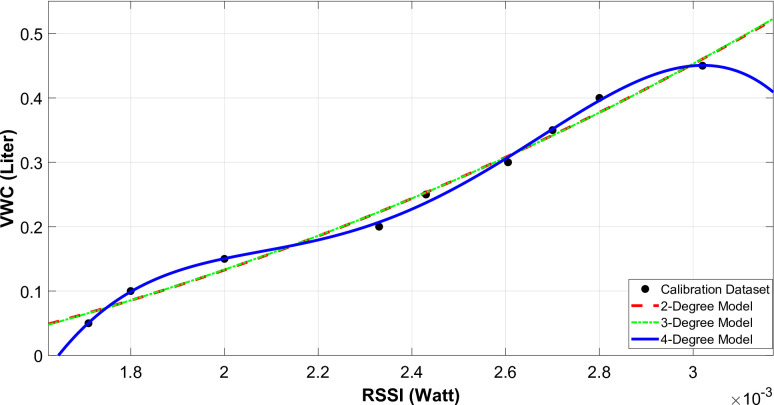
Comparative performance analysis of 2nd, 3rd, and 4th polynomial regression models.

To thoroughly validate the mathematical selection of the fourth-order polynomial mapping function and address concerns regarding potential overfitting, a comparative statistical analysis was performed against second- and third-order models using the empirical sand soil calibration dataset as demonstrated in Fig. 10. The second-order (quadratic) model exhibits severe underfitting and a suboptimal adjusted coefficient of determination $\:{R}^{2}=91.26\:\%$. This performance drop is most pronounced near the VWC saturation plateau, where the quadratic curve fails to track the sharp non-linear flattening of the RSSI signal at higher moisture levels. While the third-order model reduces the error with $\:{R}^{2}=91.74\:\%$, the fourth-order polynomial yields the most optimal performance, achieving a superior $\:{R}^{2}=99.76\:\%$.

### Model training and validation performance

**Fig. 11:**
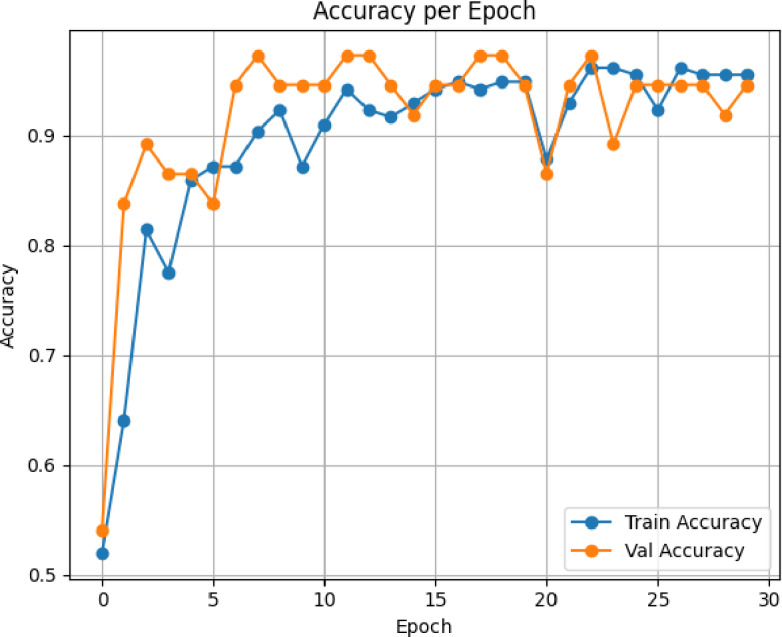
Training and validation accuracy curve of the proposed system.

**Fig. 12:**
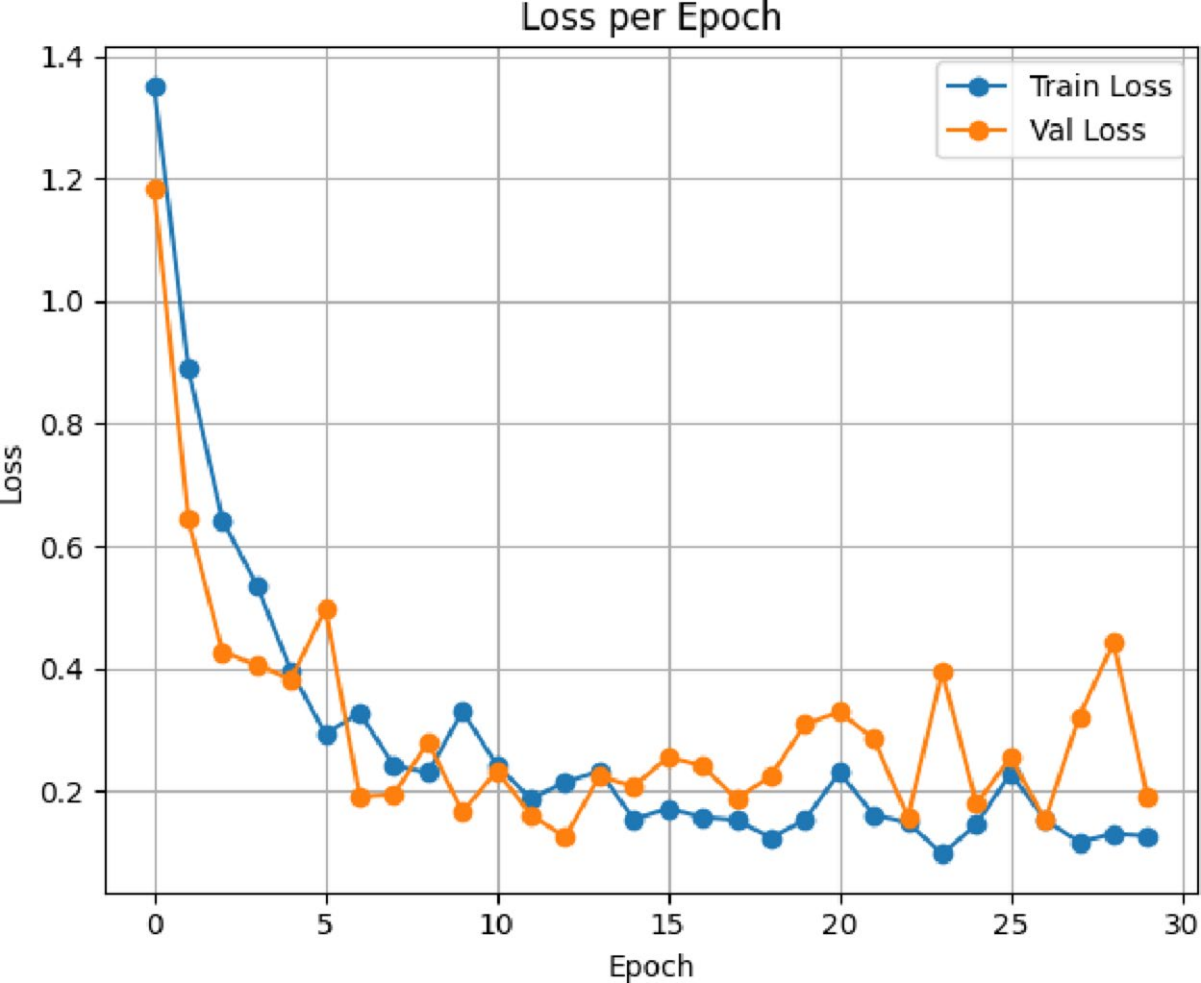
Training and validation loss curve of the proposed system.

The performance of the proposed CNN model is evaluated through training and validation phases over 30 epochs. Figure 11 illustrates the training and validation accuracy curves, demonstrating the model’s ability to learn discriminatory features effectively. An increasing trend in accuracy across both datasets confirms that the model successfully identified distinct patterns associated with the three soil classes. The stability of the model is further validated by comparing training and validation loss curves as depicted in Fig. 12. The concurrent decrease in both training and validation loss validates that the model effectively minimized prediction errors without undergoing overfitting. The close proximity between training and validation accuracy metrics throughout the training epochs suggests robust generalization, ensuring that the model maintains high reliability when processing unseen soil samples.

**Fig. 13:**
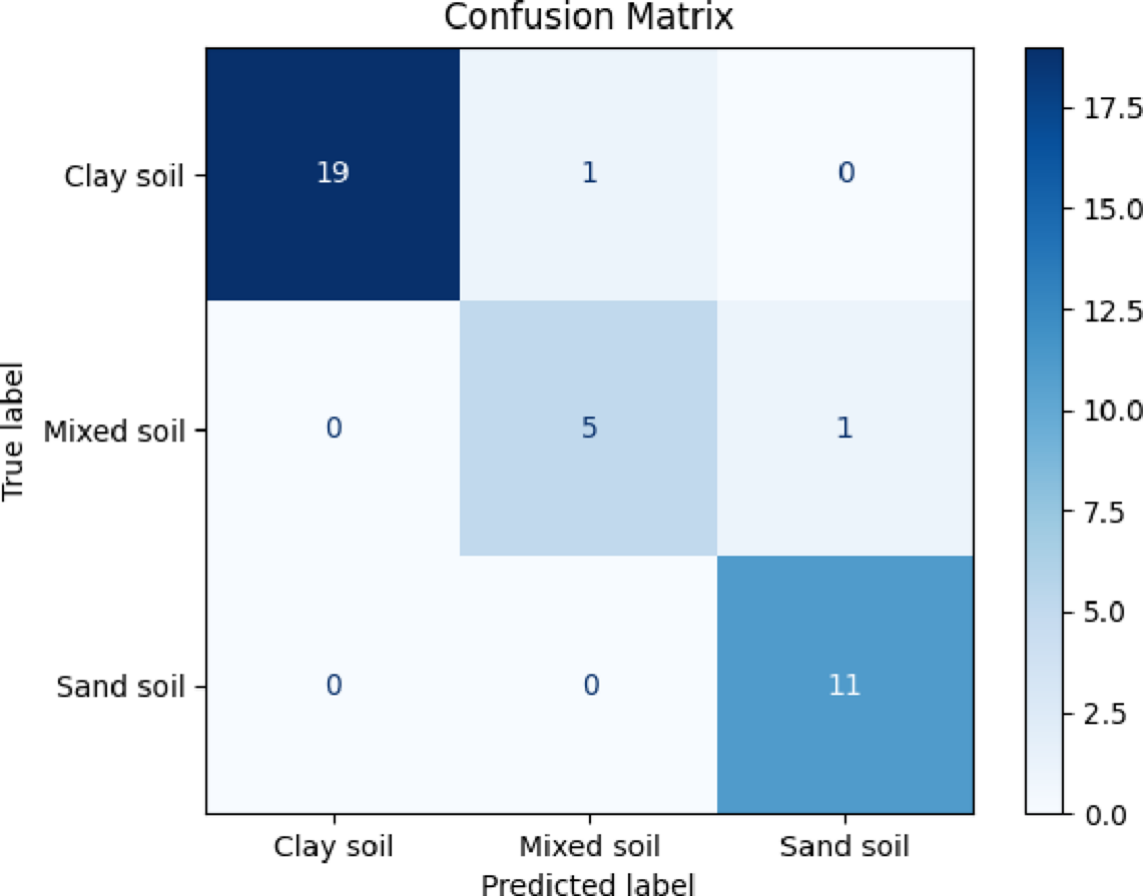
Confusion Matrix of the proposed system.

To quantitatively assess the model’s classification performance, a confusion matrix is generated as illustrated in Fig. 13. The results demonstrate superior classification capability, with overall accuracy ranging between 94% and 98% and F1-scores consistently exceeding 0.94. The confusion matrix reveals the following performance dynamics:

- *Sand Class*: The model achieved perfect classification accuracy for the sandy soil samples, likely due to the distinct coarse texture and dielectric signature of sand.
- *Clay and Mixed Soil Classes*: Minimal misclassifications were observed between clay and mixed soil samples. This minor overlap is attributed to the inherent similarities in the fine-particle composition and moisture-retention characteristics shared between these two classes.

Despite the presence of class imbalance, the model maintained high precision and recall, yielding performance metrics comparable to current state-of-the-art CNN-based classification models. These results confirm the model’s efficacy in accurately identifying soil types, which is a critical prerequisite for selecting the appropriate 4th-degree polynomial regression coefficients for accurate VWC estimation.

### Evaluation of the System Performance

To validate the accuracy of the proposed system, five pre-prepared samples (for each soil type) with known actual water contents (0.05, 0.15, 0.25, 0.35 and 0.45 L) are measured and compared with the actual values as depicted in Tables 6 and 7, and 8 for sand, clay, and mixed soil, respectively. The comparative results show high average accuracies ($\:99.8\%$ for sand, $\:99.7\mathrm{\%}$ for clay, and $\:99.4\mathrm{\%}$ for mixed soil).

**Table 6:** Actual VWC, measured VWC, and measured RSSI for sand soil.

Actual VWC	0.05	0.15	0.25	0.35	0.45
Measured VWC	0.0499	0.1497	0.2495	0.3493	0.4485
Measured RSSI	1.492	1.696	1.891	2.17	2.392
Average accuracy	$\:99.8\%$				

**Table 7:** Actual VWC, measured VWC, and measured RSSI for clay soil.

Actual VWC	0.05	0.15	0.25	0.35	0.45
Measured VWC	0.0496	0.14955	0.24925	0.34895	0.447
Measured RSSI	1.9	2.1	2.36	2.7	3
Average accuracy	$\:99.7\mathrm{\%}$				

**Table 8:** Actual VWC, measured VWC, and measured RSSI for mixed soil.

Actual VWC	0.05	0.15	0.25	0.35	0.45
Measured VWC	0.0507	0.1491	0.2485	0.3479	0.445
Measured RSSI	1.697	2.01	2.412	2.675	2.97
Average accuracy	$\:99.4\mathrm{\%}$				

### Comparison with the Standard Gravimetric (oven-drying)

To ensure the accuracy of the proposed system, the gravimetric (oven-drying) method is utilized as the reference technique to establish ground-truth moisture content. This method is universally recognized as the standard laboratory protocol, providing the high precision required to calibrate indirect sensing systems via direct mass-based determination. Notwithstanding its inherent destructive nature and temporal requirements, the gravimetric approach remains the definitive benchmark for validation. The experimental protocol for each soil sample was as follows:

#### Step (1) wet mass measurement

The initial mass of the wet soil sample $\:\left({m}_{w}\right)$ is measured.

#### Step (2) oven drying

The sample is placed in a laboratory oven at a temperature of 105 °C ± 2 °C for 24 h (until constant mass).

#### Step (3) dry mass measurement

After drying, the sample is weighed again, where $\:\left({m}_{d}\right)$ denotes the mass of dry soil.

Step (4) calculate the gravimetric moisture content $\:{(\theta\:}_{g})$ according to (16) :

16 $$\:{\theta\:}_{g}=\frac{{m}_{w}-{m}_{d}}{{m}_{d}}$$

Step (5) determine the $\:{\theta\:}_{v}={\theta\:}_{g}\cdot\:{\rho\:}_{b}$, where $\:{\rho\:}_{b}$ is the soil bulk density (g/cm³), calculated directly using the dry mass ($\:{m}_{d}$) from Step 3 and the known fixed volume (* V*) of the rigid sampling cylinder that strictly confined the soil to prevent swelling. Accordingly, the the soil bulk density is calculated as follows $\:({\rho\:}_{b}\:=\:{m}_{d}\:/\:V)$.

VWC for three prepared samples (one sample for each soil type) is determined gravimetrically following standardized procedures using a forced-convection laboratory oven (Model TR 240, Nabertherm GmbH). Soil samples are dried at 105⸰ C for 24 hours to ensure the complete removal of pore water. Sample masses are measured using an analytical balance with a precision of $\:\pm\:\:0.01$ g. Gravimetric water content $\:\left({{\uptheta\:}}_{\mathrm{g}}\right)$ for each sample separately is calculated as the mass of water lost relative to the dry soil mass. Subsequently, VWC for the three samples are derived by multiplying $\:\left({{\uptheta\:}}_{\mathrm{g}}\right)$ by the measured soil bulk density $\:{({\uprho\:}}_{\mathrm{b}})$. Table 9 presents a comparison of VWC measurements obtained from the proposed system and the gravimetric reference method across diverse soil textures. The results demonstrate that the proposed system achieves accuracy commensurate with the standard gravimetric procedure. Furthermore, while the gravimetric method necessitates a 24-hour drying duration to provide results, the proposed system enables instantaneous data acquisition, offering a significant advantage in temporal efficiency for soil moisture monitoring. To contextualize the validation performance presented in Table 9, a distinction must be made between ‘Target Nominal VWC’ and ‘Oven-Dried VWC’. The former represents the strictly controlled volumetric water ratio introduced during initial sample preparation, while the latter serves as the destructive gravimetric reference baseline. Minor discrepancies between the physical oven-dried reference and nominal values are attributed to unavoidable localized variations in dry bulk density $\:{({\uprho\:}}_{\mathrm{b}})$ during manual compaction. Crucially, because the 2.4 GHz microwave sensor captures an integrated spatial average across its entire antenna beamwidth footprint, the proposed method effectively smooths out these localized material anomalies, yielding an optimization curve that bridges minor experimental discrepancies between preparation targets and local physical measurements.

**Table 9:** Comparison between the VWC measured by the Proposed system and by Standard Gravimetric (oven-drying) Method for different soil types.

Soil type Measuring techniqe	sand soil	clay soil	Mixed soil
Target Nominal VWC $\:\left({V}_{w}/{V}_{t}\right)$	0.25	0.25	0.25
VWC measured by Proposed system	0.2495	0.24925	0.2485
VWC measured by Standard Gravimetric Method	0.251	0.2488	0.2476

## Conclusion

This study developed and validated a non-destructive framework for real-time soil water content estimation by integrating 2.4 GHz microwave/RF sensing with edge-based machine learning. The proposed system combines RSSI-based electromagnetic measurement with CNN-based soil classification to enable adaptive selection of soil-specific fourth-degree polynomial regression models for sand, clay, and mixed soil. The calibration results demonstrated a strong nonlinear relationship between RSSI response and water content, with coefficients of determination exceeding 0.95 across the tested soil types. The CNN classifier achieved high classification performance, with overall accuracy ranging from 94% to 98% and F1-scores exceeding 0.94, confirming its suitability for supporting texture-dependent model selection.

The validation results further confirmed the capability of the proposed framework to estimate soil water content with high agreement relative to the prepared reference values. Average prediction accuracies of approximately 99.8%, 99.7%, and 99.4% were obtained for sand, clay, and mixed soil, respectively. Comparison with the gravimetric oven-drying reference method also showed close agreement across the three soil textures, while reducing the measurement time from approximately 24 h to near-real-time acquisition. These findings indicate that coupling soil classification with texture-specific RSSI–moisture calibration can improve the adaptability and practical applicability of electromagnetic soil moisture estimation.

### Field Limitations

Despite these promising results, several limitations should be acknowledged. First, the experimental validation was conducted under controlled laboratory conditions using a limited number of soil textures and hydration levels. Therefore, the current calibration models may not fully represent the variability encountered under field conditions, where soil compaction, surface roughness, salinity, temperature, organic matter, mineral composition, and heterogeneous layering can affect electromagnetic and optical responses.

Specifically, variable surface roughness and soil compaction directly alter bulk density and surface scattering properties, which can introduce non-linear offsets into the RF attenuation curve. Furthermore, elevated soil salinity increases ionic conductivity, causing steeper signal attenuation that could compress the system’s dynamic sensing range. Rapid ambient temperature shifts may also induce minor hardware drift, while subsurface soil layering poses a distinct physical boundary condition; in highly stratified soils, surface visual features decouple from deep volumetric moisture content, marking a clear operational boundary for the vision subsystem.

Second, the fourth-degree polynomial models were derived from soil-specific calibration datasets; consequently, their direct transferability to other soils requires additional validation, and future iterations will need to incorporate bulk density and electrical conductivity as secondary corrective inputs. Third, the CNN classification model was evaluated using predefined soil classes, and its robustness under variable illumination, soil surface conditions, and mixed-texture field samples remains to be further examined. However, it should be noted that the system’s hardware design—specifically the light-shielded structural enclosure and the conversion of imagery to single-channel grayscale with adaptive histogram equalization—was engineered precisely to preemptively mitigate such real-world environmental lighting variations and surface color shifts.

### Future work

Future work should focus on expanding the experimental dataset to include a wider range of soil textures, densities, salinity levels, organic matter contents, and environmental conditions. Field-scale validation is also required to assess long-term stability, sensor repeatability, and model robustness under natural moisture dynamics. Further development may include uncertainty quantification, comparison with lower- and higher-order regression models, integration of additional electromagnetic features, and deployment through IoT-enabled monitoring networks. These improvements would strengthen the generalizability of the framework and support its application in geotechnical monitoring, precision agriculture, and environmental water-resource management.

## Data Availability

Data will be made available on request.
